# Impairment of motivation in patients with schizophrenia and the development of a program for their psychosocial treatment

**DOI:** 10.1192/j.eurpsy.2021.1396

**Published:** 2021-08-13

**Authors:** V. Mitikhin, V. Yastrebova, A. Nochevkina, L. Alieva

**Affiliations:** Department Of Mental Health Support Systems Research Centre, Mental Health Research Centre, Moscow, Russian Federation

**Keywords:** motivation, schizophrénia, psychosocial, Treatment

## Abstract

**Introduction:**

Reduced motivation in schizophrenia is expressed is as the reduction of activity and social functioning.

**Objectives:**

Assessment of motivation in patients with schizophrenia and development to their psychosocial treatment.

**Methods:**

Clinical, psychometric (URICA, PSP, PANSS), statistical. Included 100 patients diagnosed with schisophrenia F.20-29(ICD-10) with various levels of deficit.

**Results:**

The analysis showed the connection between the intensity of negative disorders of the patients with the level of their motivations: in the group of patients with severe deficiency (pseudoorganic), a decrease in the level of motivation was found: the score of the «Precontemplation» stage of the URICA scale (56.20 ± 9.29) was higher (P <0.001). Patients with moderate deficit (diminished schizoid) changes were distinguished by high motivation, high scores on the «Action» (49.34 ± 8.22, P <0.005) and «Maintenance» scale (52.43 ± 10.51, P <0.005). A negative correlation was established between the indicators of patient motivation and the PANSS scale: will disorders (r = -0.75, P <0.01), social withdrawal (r = -0.64, P <0.01), blunted affect (r = -0.62, P<0.005), etc. High positive correlation found between motivation rating and PSP scores in socially activity (r = 0,74, P <0,005) and social relationships (r = 0,65, P <0,01). We have developed a comprehensive program of psychosocial treatment, including compliance therapy, motivational, cognitive and social skills training, destigmatization actions and an assessment of its effectiveness.

**Conclusions:**

The developed rehabilitation program showed high efficiency: increasing motivation, reducing self-stigmatization, developing communication skills, improving social functioning and cognitive sphere in patients with schizophrenia.

